# mRNA Processing Factor CstF-50 and Ubiquitin Escort Factor p97 Are BRCA1/BARD1 Cofactors Involved in Chromatin Remodeling during the DNA Damage Response

**DOI:** 10.1128/MCB.00364-17

**Published:** 2018-01-29

**Authors:** Danae Fonseca, Jorge Baquero, Michael R. Murphy, Gamage Aruggoda, Sophia Varriano, Carmen Sapienza, Oksana Mashadova, Shadaqur Rahman, Frida E. Kleiman

**Affiliations:** a Chemistry Department, Hunter College and Biochemistry Program, Graduate Center, City University of New York, New York, New York, USA; b Fels Institute for Cancer Research and Molecular Biology, Temple University School of Medicine, Philadelphia, Pennsylvania, USA

**Keywords:** BRCA1/BARD1, CstF-50, DNA damage response, RNA polymerase II, chromatin remodeling, histones, p97

## Abstract

The cellular response to DNA damage is an intricate mechanism that involves the interplay among several pathways. In this study, we provide evidence of the roles of the polyadenylation factor cleavage stimulation factor 50 (CstF-50) and the ubiquitin (Ub) escort factor p97 as cofactors of BRCA1/BARD1 E3 Ub ligase, facilitating chromatin remodeling during the DNA damage response (DDR). CstF-50 and p97 formed complexes with BRCA1/BARD1, Ub, and some BRCA1/BARD1 substrates, such as RNA polymerase (RNAP) II and histones. Furthermore, CstF-50 and p97 had an additive effect on the activation of the ubiquitination of these BRCA1/BARD1 substrates during DDR. Importantly, as a result of these functional interactions, BRCA1/BARD1/CstF-50/p97 had a specific effect on the chromatin structure of genes that were differentially expressed. This study provides new insights into the roles of RNA processing, BRCA1/BARD1, the Ub pathway, and chromatin structure during DDR.

## INTRODUCTION

All eukaryotes have evolved an intricate network of surveillance mechanisms or DNA damage response (DDR) processes to maintain genomic integrity against a variety of cellular insults. DDR includes not only repair mechanisms but also regulation of the cell cycle, transcription (1, 2), and mRNA processing (3). Ubiquitination regulates many cellular processes, but its potential functional connection with mRNA 3′ processing and DNA damage has not been completely characterized. BRCA1 and BRCA1-associated RING domain protein 1 (BARD1) form a heterodimer that exhibits E3 ubiquitin (Ub) ligase activity that is quickly recruited to DNA lesions during DDR (4). The BRCA1/BARD1 complex plays a role in the UV-induced inhibition of mRNA 3′ processing (5), resulting in the transient decrease of the cellular levels of polyadenylated transcripts. mRNA 3′ processing can be repressed after DNA damage as a result of the interaction of BRCA1/BARD1 with cleavage stimulation factor 50 (CstF-50) (5–7) and the proteasome-mediated degradation of RNA polymerase (RNAP) II, which is an activator of the cleavage step of the polyadenylation reaction (6). In fact, the largest subunit of RNAP IIO, which functions in elongation, but not that of RNAP IIA, the form engaged in promoters, is a BRCA1/BARD1 substrate (6, 8). Consistent with this, the small interfering RNA (siRNA)-mediated knockdown of BRCA1/BARD1 in HeLa cells reduces the UV-induced ubiquitination and degradation of RNAP II (6). Interestingly, either the depletion of CstF in UV-treated cells (9) or the expression of BARD1 phosphorylation mutants impaired in interacting with CstF-50 (10) abrogated RNAP II ubiquitination, suggesting a role of CstF in RNAP II ubiquitination.

Other BRCA1/BARD1 Ub ligase targets are histones H_2_A (11–13) and H_2_B (11, 14). BRCA1/BARD1 can regulate chromatin structure by binding to satellite DNA regions and ubiquitinating histone H_2_A (Ub-H_2_A) (15). Cells deficient in BRCA1 show impaired heterochromatin integrity that causes the disruption of gene silencing at tandem-repeat DNA regions due to the loss of Ub-H_2_A (15). BRCA1/BARD1 Ub ligase participates in chromatin remodeling, which includes H_2_A ubiquitination, allowing repair of double-strand breaks (DSB) (13). Interestingly, it has also been shown that UV treatment induces monoubiquitination of H_2_A (16), resulting in the eviction of Ub-H_2_A from UV-damaged chromatin (17).

BRCA1 interacts physically with p97, a weak ATPase with escort protein functions in the Ub pathways involved in multiple cellular events, in the nuclei of living cells (18, 19). It functions in binding ubiquitinated substrates, segregating them from their binding partners, and escorting them to the proteasome, as well as in controlling the degree of ubiquitination of the substrate by interacting with cofactors (20). p97 is involved in chromatin remodeling, which allows the recruitment of BRCA1 to DNA damage sites (21). p97 is one of the most abundant proteins in the nucleoplasm, and it has been implicated in DDR. For example, p97 overexpression abrogates genome instability that results from an excess of DNA damage sensors and repair initiators at damaged sites (22). p97 also plays a role in UV-induced RNAP II degradation in mammalian cells, resulting in the extraction of ubiquitinated RNAP II from chromatin (23). Although p97 interaction with BRCA1 and its role in RNAP II ubiquitination suggest a role of p97 in BRCA1/BARD1-mediated ubiquitination, its function in this pathway remains unknown.

Despite our understanding of the biochemistry of the BRCA1/BARD1 E3 ligase, we know very little of the mechanism by which its cellular targets are chosen, how its E3 Ub ligase activity is regulated to activate its function, which cofactors are involved in this regulation, and how the substrates are recruited to DNA damage sites during DDR. Here, we provide evidence that the mRNA processing factor CstF-50 and the Ub escort factor p97 are BRCA1/BARD1 cofactors involved in chromatin remodeling of genes differently transcribed during DDR. We show that CstF-50 can interact directly not only with BRCA1/BARD1 but also with Ub, the escort factor p97, and some BRCA1/BARD1 substrates, such as RNAP II, H_2_A, and H_2_B. Also, CstF-50, together with p97, activates BRCA1/BARD1-dependent H_2_A and H_2_B monoubiquitination, RNAP II polyubiquitination, and BRCA1/BARD1 autoubiquitination. CstF-50 and p97 are BRCA1/BARD1 cofactors that participate in the ubiquitination of these BRCA1/BARD1 substrates during DDR. We also show that the content of monoubiquitinated H_2_B and H_2_A in the chromatin of genes with different levels of expression changes during DDR, and this is mediated by BRCA1/BARD1 and CstF-50 expression and p97 ATPase activity. Taken together, our results provide evidence that CstF-50-associated p97 regulates BRCA1/BARD1 Ub ligase activity during DDR, helping in the assembly and/or stabilization of the ubiquitination complex and affecting the chromatin structure and, hence, gene expression.

## RESULTS

### CstF-50 and p97 activate BRCA1/BARD1 Ub ligase *in vitro*.

Work from different groups has shown that the mRNA processing factor CstF-50 (5, 24, 25) and the Ub escort factor p97 (18) can interact with BRCA1/BARD1. Both CstF-50 (9) and p97 (23) play roles in the ubiquitination of RNAP II, which is an enzymatic substrate for BRCA1/BARD1 E3 Ub ligase (6, 8). CstF-50 can also bind the C-terminal domain (CTD) of RNAP II (6, 26, 27). Together, these studies suggest that CstF-50 and p97 might functionally overlap by regulating the ubiquitination of BRCA1/BARD1 substrates, such as RNAP II, and helping in either the assembly or the stabilization of the ubiquitination complex.

**FIG 1:**
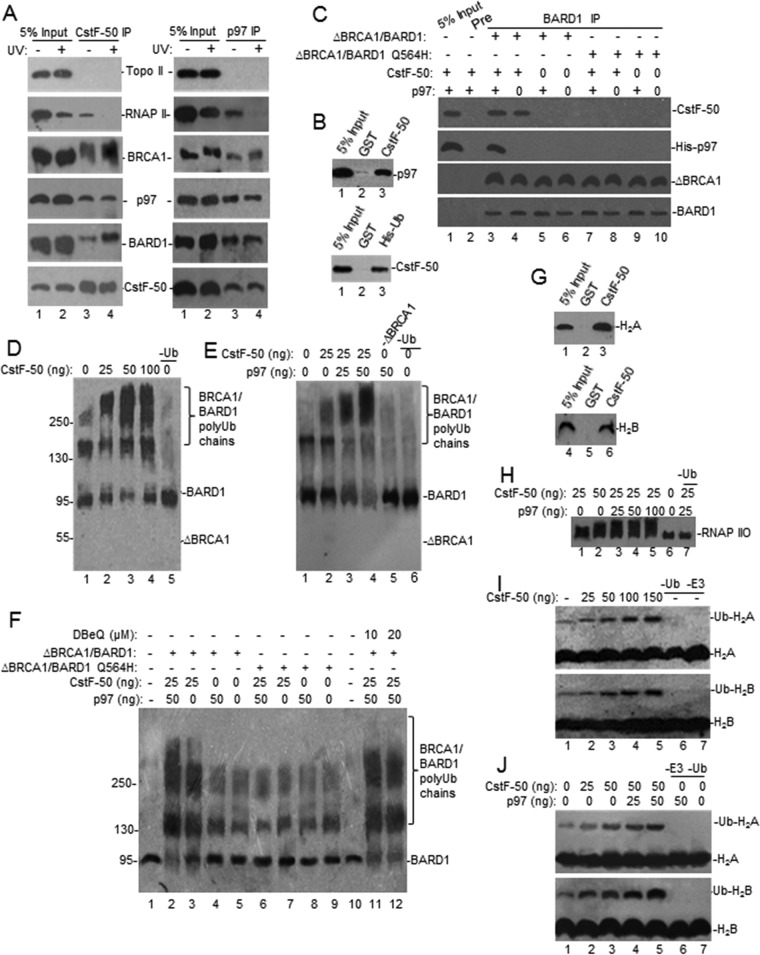
CstF-50 interacts with p97 to form a protein complex, and both can activate BRCA1/BARD1 Ub ligase activity *in vitro* in an additive manner. (A) p97, CstF-50, BRCA1, and BARD1 can form complexes in NEs of HeLa cells independently of UV treatment. Cells were exposed to UV irradiation (40 J m^−2^) and allowed to recover for 2 h before NEs were prepared. The NEs were immunoprecipitated with anti-CstF-50 (left) or anti-p97 (right). Equivalent amounts of the pellets (IP) were resolved by SDS-PAGE, and proteins were detected by immunoblotting using antibodies against the indicated proteins. Antibody against Topo II was used as a control for specificity. The positions of Topo II, RNAP II, BRCA1, BARD1, p97, and CstF-50 are indicated. Five percent of the NE used in the IP reaction is shown as input. Representative IP reactions from three independent assays are shown. (B) CstF-50 interacts directly with p97. (Top) Immobilized GST–CstF-50 or GST on glutathione beads was incubated with 1 μg of His-p97. (Bottom) Immobilized His-p97 on nickel beads was incubated with GST–CstF-50 or GST. Bound proteins were eluted, resolved by SDS-PAGE, and detected by Western blotting with anti-p97 or anti-CstF-50. Five percent His-p97 or GST–CstF-50 used in the reaction is shown as input. Recombinant proteins were treated with RNase A. Representative pulldown reactions from three independent assays are shown. (C) CstF-50 bridges the interaction between BARD1 and p97. Pulldown assays were conducted in the presence of truncated (residues 1 to 304) BRCA1 and full-length BARD1 (WT or BARD1-Q564H) heterodimer. Either ΔBRCA1/BARD1-wt or ΔBRCA1/BARD1-Q564H was incubated with GST–CstF-50 and/or His-p97. Samples were immunoprecipitated with anti-BARD1 or preimmune sera. Bound proteins were eluted, resolved by SDS-PAGE, and detected by Western blotting with CstF-50, BARD1, BRCA1, or p97 antibodies; 5% His-p97 or GST–CstF-50 used in the reaction is shown as input. (D) CstF-50 increases the autoubiquitination of the BRCA1/BARD1 heterodimer. *In vitro* ubiquitination reactions were conducted in the presence of limiting amounts (10 ng) of ΔBRCA1/BARD1-wt, E1, His-E2, His-Ub, and ATP and increasing amounts of recombinant GST–CstF-50. Samples were resolved by SDS-PAGE and immunoblotted with anti-BARD1. Representative autoubiquitination reactions from three independent assays are shown. (E) p97 further increases CstF-50-mediated activation of BRCA1/BARD1 autoubiquitination. *In vitro* ubiquitination reactions were carried out as for panel D but using limiting amounts of GST–CstF-50 (25 ng) and increasing amounts of His-p97. Samples were resolved by SDS-PAGE and immunoblotted with anti-BARD1. Representative autoubiquitination reactions from three independent assays are shown. (F) CstF-50/p97-dependent enhancement of BRCA1/BARD1 E3 Ub ligase activity depends on the ability of CstF-50 to bind the heterodimer. Autoubiquitination assays were carried out as for panel D but also including ΔBRCA1/BARD1-Q564H and increasing amounts of the p97 inhibitor DBeQ. (G) CstF-50 interacts directly with H_2_A (top) and H_2_B (bottom). Immobilized GST–CstF-50 on glutathione-agarose beads was incubated with commercially available H_2_A and H_2_B. Bound proteins were eluted, resolved by SDS-PAGE, and detected with anti-histones; 5% histones used in the reaction were loaded as input. Representative pulldown reactions from three independent assays are shown. (H) CstF-50 and p97 activate RNAP IIO ubiquitination by BRCA1/BARD1. *In vitro* ubiquitination reactions were performed as described for panel D in the presence of purified RNAP IIO. Proteins were detected with RNAP IIO-specific antibody (H5; Covance). Representative ubiquitination reactions from three independent assays are shown. (I) CstF-50 activates the monoubiquitination of H_2_A and H_2_B by BRCA1/BARD1. *In vitro* ubiquitination reactions were performed as described for panel D in the presence of commercially available H_2_A and H_2_B and increasing amounts of GST–CstF-50. Proteins were detected by Western blotting with anti-histones. (J) p97 further increases CstF-50-mediated activation of histone monoubiquitination by BRCA1/BARD1. *In vitro* ubiquitination reactions were performed as for panel I but using limiting amounts of GST–CstF-50 and increasing amounts of His-p97. Representative ubiquitination reactions from three independent assays are shown.

To analyze this possibility, first, we examined the physical association of CstF-50 with p97. Our endogenous-immunoprecipitation (e-IP) (Fig. 1A) and pulldown (Fig. 1B) assays indicated that CstF-50 can interact directly with p97 (Fig. 1B) and can form protein complexes with p97 in nuclear extracts (NEs) from HeLa cells under nonstress conditions and after UV treatment (40 J m^−2^) (Fig. 1A, left). CstF-50 also immunoprecipitated BRCA1 and BARD1. Consistent with previous studies, our e-IPs with CstF-50 showed the UV-induced degradation of RNAP II (6) and the UV-induced increase in CstF-50/BARD1/BRCA1 complex formation (5). The reciprocal coimmunoprecipitation (co-IP) analysis using antibodies against p97 showed no change in the interaction of p97 with CstF-50, BRCA1, and BARD1 under DNA-damaging conditions (Fig. 1A, right). Both CstF-50 and p97 interacted with RNAP II, which is one of the BRCA1/BARD1 substrates (6, 8). As the samples were analyzed under PAGE-denaturing conditions, our results showed the direct and noncovalent association of CstF-50 with Ub (see Fig. S1A and B in the supplemental material), one of the CstF-50 interactors detected when using yeast two-hybrid screening (9, 24). Because samples were treated with RNase A, the observed interactions were probably not due to an RNA-tethering effect.

To further analyze these complexes, we performed pulldown assays using a heterodimeric complex (ΔBRCA1/BARD1-wt) comprised of truncated BRCA1 spanning the RING domain (residues 1 to 304) and full-length BARD1 (Fig. 1C). The truncated BRCA1 does not include the p97 binding region (residues 303 to 625) (18). P97 was pulled down by BARD1 only in the presence of CstF-50, indicating that CstF-50 serves as a bridge between the two proteins (Fig. 1C). Supporting this, a tumor-associated germ line mutant of BARD1 (Q564H) with intact E3 Ub ligase activity and reduced binding to CstF-50 (5) did not interact with p97. These results indicate that p97 binds the BRCA1/BARD1/CstF-50 complex through both BRCA1 and CstF-50. Together, these experiments indicate that CstF-50 can interact directly and noncovalently not only with BRCA1/BARD1 but also with p97, Ub, and RNAP II, which is one of the BRCA1/BARD1 substrates (6, 8), suggesting that CstF-50 and p97 might function as a cofactor/scaffold for BRCA1/BARD1-mediated ubiquitination, probably helping to determine substrate specificity.

To determine whether CstF-50 and p97 play roles in regulating BRCA1/BARD1 Ub ligase activity, we performed *in vitro* ubiquitination assays using recombinant proteins (see Fig. S1C to E in the supplemental material). First, we analyzed the effect of increasing amounts of CstF-50 on the autoubiquitination of BRCA1/BARD1. The heterodimeric complex ΔBRCA1/BARD1-wt described above was incubated with E1, its cognate E2 (UbcH5c), His-Ub, and increasing amounts of glutathione *S*-transferase (GST)–CstF-50. As indicated by the low-mobility species (Fig. 1D), the heterodimer autoubiquitination increased with the amount of GST–CstF-50 added to the reaction. As expected, a decrease in unmodified BARD1 (Fig. 1D), as well as ΔBRCA1 (see Fig. S1F in the supplemental material), was observed. These results indicate that CstF-50 can enhance the autoubiquitination of BRCA1/BARD1 *in vitro*, as well as the length of the polyubiquitinated chains formed. Surprisingly, increasing amounts of p97 further activated BRCA1/BARD1 autoubiquitination in the presence of optimal amounts of CstF-50 (Fig. 1E), suggesting an additive effect between p97 and CstF-50 in activating BRCA1/BARD1 Ub ligase activity. Addition of p97 alone did not activate BRCA1/BARD1 activity. Nonspecific ubiquitination was detected by blotting with antibodies against Ub (see Fig. S1G and H in the supplemental material). No ubiquitination was detected in the absence of E2 or E3 Ub ligase (see Fig. S1I in the supplemental material), indicating that neither CstF-50 nor p97 had those functions. Using a ΔBRCA1/BARD1-Q564H mutant with intact E3 Ub ligase activity and reduced binding to CstF-50 (5), we determined that the CstF-50/p97 synergistic activation of BRCA1/BARD1 depends on the ability of CstF-50 to bind BARD1 (Fig. 1F, lanes 6 to 9). By adding *N*_2_,*N*_4_-dibenzylquinazoline-2,4-diamine (DBeQ), an inhibitor of p97 ATPase activity (28), to the ubiquitination reaction mixture, we showed that p97's ATPase activity was not necessary to further increase the CstF-50-mediated activation of BRCA1/BARD1 Ub ligase activity (Fig. 1F, lanes 11 and 12). As p97 cannot bind ΔBRCA1 (18), the synergistic effect observed can be attained only through its ability to bind CstF-50 (Fig. 1C). While a bacterial contaminant might be an explanation for the increase in ligase activity observed after p97 addition, our His-p97 sample seemed quite pure (see Fig. S1D in the supplemental material). While further experiments are necessary to determine the details of this pathway, these data indicate that CstF-50 and p97 are cofactors for BRCA1/BARD1-mediated autoubiquitination and that p97 plays a nonenzymatic additive role in this reaction.

Next, we tested whether CstF-50 and p97 can activate BRCA1/BARD1 in *trans*-ubiquitination reactions using known BRCA1/BARD1 substrates, such as RNAP II, H_2_A, and H_2_B (15, 29). We previously showed the interaction of CstF-50 with both BRCA1/BARD1 (5, 24) and one of its substrates, RNAP II (6, 9). Extending those studies here, using pulldown assays, we show that CstF-50 can interact directly with both H_2_A and H_2_B (Fig. 1G). Then, we analyzed the role of CstF-50 in BRCA1/BARD1-mediated ubiquitination of RNAP IIO and commercially available H_2_A and H_2_B in *in vitro* ubiquitination reactions, as shown in Fig. 1D. Previously, we and others have shown that RNAP IIO, which functions in transcription elongation, is an enzymatic substrate for BRCA1/BARD1 E3 Ub ligase (6, 8) and that depletion of CstF in DT40 cells reduces UV-induced ubiquitination of RNAP IIO (9). We also showed that RNAP IIO ubiquitination was completely dependent on the presence of recombinant BRCA1/BARD1 and that the tumor-associated BRCA1 mutation C61G, which disrupts the RING domain, abolished RNAP IIO modification (6). Here, ubiquitination of elongating RNAP IIO was examined using antibodies directed against the Ser 2-phosphorylated CTD epitope of RNAP II (H5) (Fig. 1H). As indicated by the low-mobility species, BRCA1/BARD1-mediated ubiquitination of RNAP IIO increased when CstF-50 was added to the reaction mixture (Fig. 1H). p97 further activated RNAP IIO polyubiquitination by BRCA1/BARD1 in the presence of CstF-50, indicating that p97 and CstF-50 cooperate in the activation of RNAP IIO ubiquitination by BRCA1/BARD1 (Fig. 1H). Interestingly, CstF-50 also increased the levels of H_2_A and H_2_B monoubiquitination in these *in vitro* ubiquitination reactions (Fig. 1I). As in the BRCA1/BARD1 autoubiquitination reactions (Fig. 1E), p97 further activated H_2_A and H_2_B monoubiquitination by BRCA1/BARD1 in the presence of CstF-50 (Fig. 1J; see Fig. S1J in the supplemental material). Together, these results indicate that CstF-50 and p97 had an additive effect on the activation of the ubiquitination of these BRCA1/BARD1 substrates.

### CstF-50 and p97 can activate BRCA1/BARD1-mediated monoubiquitination of H_2_A and H_2_B and polyubiquitination of RNAP IIO in soluble nuclear extracts from HeLa cells.

**FIG 2:**
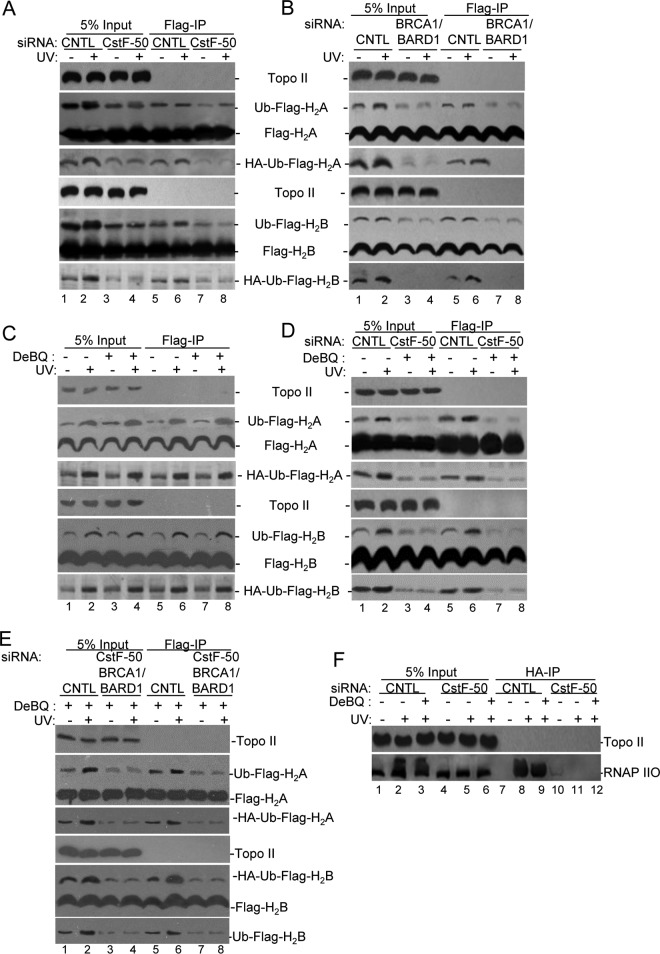
Monoubiquitination of histones H_2_A and H_2_B and polyubiquitination of RNAP IIO by BRCA1/BARD1 are affected by the functional interaction of CstF-50 and p97. CstF-50 and p97 activate UV-induced monoubiquitination of histones. (A to E) HeLa cells were transfected with siRNAs for either control (CNTL), CstF-50 (A and D), or BRCA1/BARD1 (B) or concomitantly for BRCA1/BARD1/CstF-50 (E). The cells were also transfected with HA-Ub and either FLAG-H_2_A or FLAG-H_2_B constructs. The cells were treated with UV (40 J m^−2^) and allowed to recover for 2 h before NEs were prepared. (C to E) Alternatively, cells were also treated with DBeQ (10 μM) during the 2-h recovery. Soluble NEs were immunoprecipitated with anti-FLAG M2 magnetic beads (Sigma), followed by Western blotting with the indicated antibodies. Antibody against Topo II was used as a control; 5% of the cell extracts used in the IP reaction is shown as input. Representative IP reactions from three independent assays are shown. (F) CstF-50 and p97 activate UV-induced polyubiquitination of RNAP IIO. HeLa cells were transfected with either control or CstF-50 siRNAs concomitantly with an HA-Ub construct. The cells were treated with UV (40 J m^−2^). The proteasomal inhibitor MG132 (2 μM) and DBeQ (10 μM) were added to the cells immediately after exposure to UV light, and the cells were allowed to recover for 2 h before soluble NEs were prepared. The soluble NEs were immunoprecipitated with anti-HA–agarose beads, and equivalent amounts of the pellets (IP) were analyzed by immunoblotting using antibodies against Topo II and RNAP IIO (H5). Antibody against Topo II was used as a control; 5% of the extracts used in the IP reaction are shown as input. Representative IP reactions from three independent assays are shown.

Extending these studies, we analyzed the role of CstF-50 in RNAP IIO, H_2_A, and H_2_B ubiquitination by using soluble NEs from HeLa cells transfected not only with hemagglutinin (HA)-tagged Ub but also with either Flag-tagged H_2_A or H_2_B expression vectors. These cells were concomitantly transfected with control, CstF-50, and/or BRCA1/BARD1 siRNAs and exposed to DNA-damaging conditions. Soluble fractions of the NEs were prepared, followed by immunoprecipitation with FLAG antibodies (Fig. 2A to E). The knockdown efficiencies are shown in Fig. S2B in the supplemental material. As previous work has shown the lack of specificity in the ubiquitination of nucleosome-free histones (29), it is important to highlight that these experiments with soluble NEs were designed to measure and characterize general ligase activity rather than as an assay of a specific target. Also, these assays allowed us to analyze histone modifications without considering their eviction from chromatin as a consequence of their ubiquitination. Consistent with previous reports (17, 30, 31), our results show a UV-induced increase in both H_2_A and H_2_B monoubiquitination. Interestingly, siRNA-mediated depletion of CstF-50 (∼80%) decreased the monoubiquitination of H_2_A and H_2_B independently of UV treatment, indicating that CstF-50 is an activator of this reaction (Fig. 2A). Western blot analysis with HA antibodies of Flag-immunoprecipitated samples confirmed the role of CstF-50 in histone monoubiquitination. No changes in the levels of total H_2_A and H_2_B were detected after either UV treatment or CstF-50 depletion. When we performed the simultaneous siRNA-mediated knockdown of BRCA1 and BARD1 (∼90%, as described in reference 6) a decrease in the monoubiquitination of H_2_A and H_2_B was also detected (Fig. 2B), suggesting that CstF-50 and BRCA1/BARD1 are parts of the same ubiquitination pathway. Consistent with the *in vitro* studies shown in Fig. 1F, treatment of cells with DBeQ, an inhibitor of the ATPase activity of p97 (28), did not alter the monoubiquitination levels of H_2_A and H_2_B (Fig. 2C). This is consistent with the fact that inhibition of p97 segregase activity did not have an effect on H_2_A and H_2_B modification (Fig. 1F). However, when CstF-50-depleted cells were treated with DBeQ and UV (Fig. 2D), inhibition of p97 ATPase activity further decreased the levels of monoubiquitinated H_2_A and H_2_B below those observed with CstF-50 depletion (compare Fig. 2A and D). This supports a nonenzymatic additive role of p97 in the E3 Ub ligase activity of BRCA1/BARD1. Analysis of soluble fractions of NEs from cells depleted of BRCA1/BARD1/CstF-50 (triple knockdown) and treated with DBeQ showed the same effect as the single (CstF-50) or double (BRCA1/BARD1) knockdowns (compare Fig. 2E with A to D), supporting the idea that these factors participate in the same ubiquitination pathway.

To further analyze the effects of CstF-50 and p97 on BRCA1/BARD1-mediated ubiquitination of RNAP IIO, we analyzed soluble NEs from HeLa cells transfected with HA-tagged Ub expression vector concomitantly with control or CstF-50 siRNAs (Fig. 2F). The proteasome inhibitor MG132 was added to the cells immediately after UV treatment to prevent degradation of RNAP II, and soluble fractions of the NEs were prepared 2 h after UV/MG132 treatment. Samples were then HA immunoprecipitated. siRNA-mediated depletion of CstF-50 resulted in a decrease in RNAP IIO ubiquitination after UV damage (Fig. 2F, compare lanes 5 and 6 to 2 and 3 and lanes 11 and 12 to 8 and 9). Interestingly, the effect of CstF-50 depletion on RNAP IIO ubiquitination was similar to the one observed in BRCA1/BARD1-depleted cells (6), indicating one more time that CstF-50 and BRCA1/BARD1 are parts of the same UV-induced ubiquitination pathway. When the transfected cells described above were treated with DBeQ (10 μM) to inhibit p97 (28), a decrease in the levels of ubiquitinated RNAP IIO after UV treatment was observed (Fig. 2F, compare lanes 2 and 3). This decrease was even more evident when samples from the cells were immunoprecipitated with HA antibodies (Fig. 2F, compare lanes 8 and 9). We did not detect any RNAP IIO immunoprecipitated with HA antibodies under nonstress conditions (lanes 7 and 10) and in samples from CstF-50 depleted cells (Fig. 2F, lanes 11 and 12). Consistent with previous observations (9), our results indicate that CstF-50 is necessary for UV-induced ubiquitination of RNAP II, which is a substrate of BRCA1/BARD1 (6, 8). Importantly, inhibition of p97 activity decreased the levels of UV-induced ubiquitination of RNAP IIO, offering an alternative mechanism for the previously described role of p97 in RNAP II ubiquitination (23).

Together, these experiments indicate that CstF-50 and p97 can activate the BRCA1/BARD1-mediated monoubiquitination of H_2_A and H_2_B and polyubiquitination of RNAP IIO. Importantly, while several inhibitors of BRCA1/BARD1 activity have been described, such as UBXN1 (32) and CDK2 (33), to our knowledge, this is the first report of an activator for BRCA1/BARD1 E3 Ub ligase activity.

### CstF-50, p97, and BRCA1/BARD1 can regulate the content of monoubiquitinated H_2_A and H_2_B in genome-wide chromatin during DDR.

**FIG 3:**
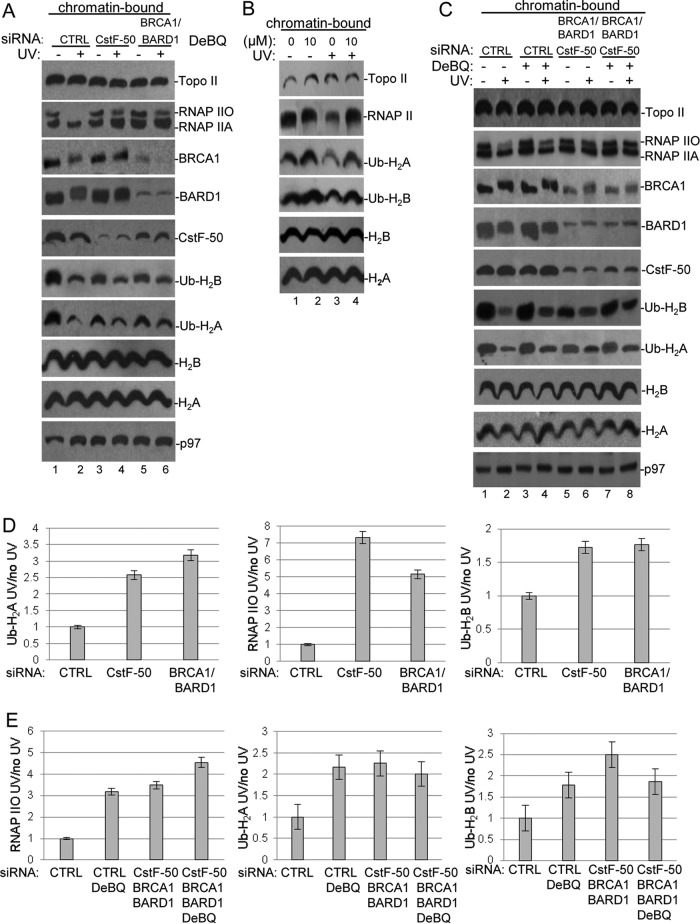
Knockdown of the BRCA1/BARD1/CstF-50/p97 complex induces changes in the localization of some of their substrates upon UV treatment in chromatin-bound fractions. (A) Chromatin-bound fractions were prepared from HeLa cells treated with control (CTRL), CstF-50, or BRCA1/BARD1 siRNA and analyzed by Western blotting with the indicated antibodies. The cells were also exposed to UV treatment (40 J m^−2^) and allowed to recover for 2 h. (B) Chromatin-bound fractions from HeLa cells treated with UV irradiation (40 J m^−2^) and the p97 inhibitor DBeQ (10 μM) during the 2-h recovery were analyzed by immunoblotting with the indicated antibodies. (C) Chromatin-bound fractions were prepared from cells treated with control and BRCA1/BARD1/CstF-50 siRNAs. The cells were also exposed to UV (40 J m^−2^) and DBeQ (10 μM) treatment during the 2-h recovery. Samples were analyzed by Western blotting with the indicated antibodies. (A to C) Representative Western blots from three independent assays are shown. (D and E) Quantification of the blots shown in panels A and C, respectively. Error bars represent the standard deviations derived from three independent experiments.

To determine the effects of BRCA1/BARD1, CstF-50, and p97 on the content of Ub-H_2_A and Ub-H_2_B in the context of genome-wide chromatin, we analyzed the effects of CstF-50 and BRCA1/BARD1 depletion and DBeQ/UV treatment on chromatin-bound fractions from HeLa cells by Western blotting (Fig. 3). Loading controls for each cellular fraction were analyzed with specific antibodies (see Fig. S2A in the supplemental material). Our results indicated that BRCA1, BARD1, and CstF-50, as well as RNAP II, were present in the chromatin-bound fractions. As expected, H_2_A, H_2_B, and their modified isoforms were also detected in the fractions. After UV treatment, the levels of RNAP IIO, Ub-H_2_A, and Ub-H_2_B decreased in chromatin-bound fractions from control cells (Fig. 3A to C). These observations are consistent with the previously described UV-induced displacement of Ub-H_2_A and Ub-H_2_B from the chromatin (17, 34–36) and the UV-induced degradation of RNAP II (6, 8). After UV treatment, no evident changes were observed in the levels of unmodified histones. A decrease in the levels of CstF-50 in chromatin-bound fractions was observed when BRCA1/BARD1 were depleted under nondamaging conditions. However, this was not evident when soluble nuclear extracts from the same cells were analyzed (see Fig. S2B in the supplemental material), suggesting that BRCA1/BARD1 might play a role in the recruitment of CstF-50 to the chromatin fraction. A decrease in the levels of BRCA1, BARD1, and CstF-50 was detected under DNA-damaging conditions, supporting previous studies that showed UV-dependent release of E3 Ub ligase from chromatin (17). Our data also indicate that p97 was recruited to the chromatin after UV treatment, in agreement with its function as a segregase that facilitates the extraction of substrate proteins from chromatin during DDR (37, 38). Neither CstF-50 nor BRCA1/BARD1 depletion affected the chromatin content of p97, indicating that p97 was recruited by its binding to either BRCA1 (18) or CstF-50, respectively. These results support the idea that the segregase might be recruited by more than one interactor. While our study (Fig. 2) and others (17, 30, 31) indicate that the levels of monoubiquitinated H_2_A and H_2_B increase in soluble nuclear extracts after UV treatment, our chromatin analysis indicated a decrease in the levels of monoubiquitinated H_2_A and H_2_B after UV treatment (Fig. 3A to C). This is consistent with the previously described eviction of Ub-H_2_A and Ub-H_2_B from UV-damaged chromatin (17, 34).

Interestingly, after UV treatment, we detected an increase in the amounts of RNAP IIO, Ub-H_2_A, and Ub-H_2_B in chromatin-bound fractions from cells depleted in BRCA1/BARD1 and CstF-50 expression (Fig. 3A; quantification is shown in panel D). These results are consistent with the previously described reduction of RNAP II degradation in cells depleted of BRCA1/BARD1 (6, 8) and CstF (9) expression under DNA-damaging conditions. Importantly, these results also suggest that cells depleted of BRCA1/BARD1 and CstF-50 expression might be defective in the UV-induced eviction of Ub-histones from chromatin (17). Supporting this, an increase in the levels of RNAP II, Ub-H_2_A, and Ub-H_2_B was observed after DBeQ treatment (Fig. 3B), indicating that ATPase activity is important for p97-mediated eviction of RNAP II, Ub-H_2_A, and Ub-H_2_B. As segregase activity occurs in the context of chromatin, the effect of DBeQ treatment was not observed when samples from soluble NEs were analyzed (Fig. 2C and D). It is important to highlight the fact that concomitant depletion of BRCA1/BARD1/CstF-50 (triple knockdown) and inhibition of p97 ATPase activity (Fig. 3B, C, and E) resulted in similar changes in the chromatin, such as retention of Ub-histones and inhibition of RNAP II degradation. Since endogenous proteins were analyzed in this set of experiments, the BRCA1/BARD1/CstF-50 depletion had a smaller effect in chromatin-bound fractions (Fig. 3) than in soluble NEs, where overexpression of tagged proteins was analyzed (Fig. 2). Although our results indicate that the functional interaction of BRCA1/BARD1/CstF-50/p97 can change the content of Ub-H_2_A and Ub-H_2_B in genome-wide chromatin after UV treatment, we cannot rule out the possibility that H_2_A and H_2_B are targeted for ubiquitination *in vivo* by more than one E3 ligase. In fact, the polycomb protein RING1B is an E3 Ub ligase that can catalyze the monoubiquitination of H_2_A after UV treatment (30, 39, 40). To determine the contribution of RING1B to the changes in RNAP II, Ub-H_2_A, and Ub-H_2_B levels in genome-wide chromatin after UV treatment, we used siRNA-mediated depletion of RING1B expression and analyzed the levels of these factors in chromatin-bound fractions after UV treatment. As shown in Fig. S2D and F in the supplemental material, after UV treatment, we observed an increase in the amount of Ub-H_2_A in chromatin-bound fractions from cells depleted of RING1B expression. RING1B expression did not significantly affect RNAP II and Ub-H_2_B content in chromatin-bound fractions after UV treatment. No significant changes in levels of Ub-H_2_A were observed after DBeQ treatment in samples from RING1B-depleted cells. Therefore, this result suggests that most likely the BRCA1/BARD1 complex and the polycomb RING1B complex have redundant roles in the UV-mediated monoubiquitination of H_2_A. Together, our data indicate that the RNAP II, Ub-H_2_A, and Ub-H_2_B content in genome-wide chromatin changes after DNA damage and that BRCA1/BARD1, p97, and CstF-50 play roles in those dynamic changes.

### BRCA1/BARD1 and CstF-50 regulate the ubiquitinated H_2_A and H_2_B content in the chromatin of differentially expressed genes during DDR.

**FIG 4:**
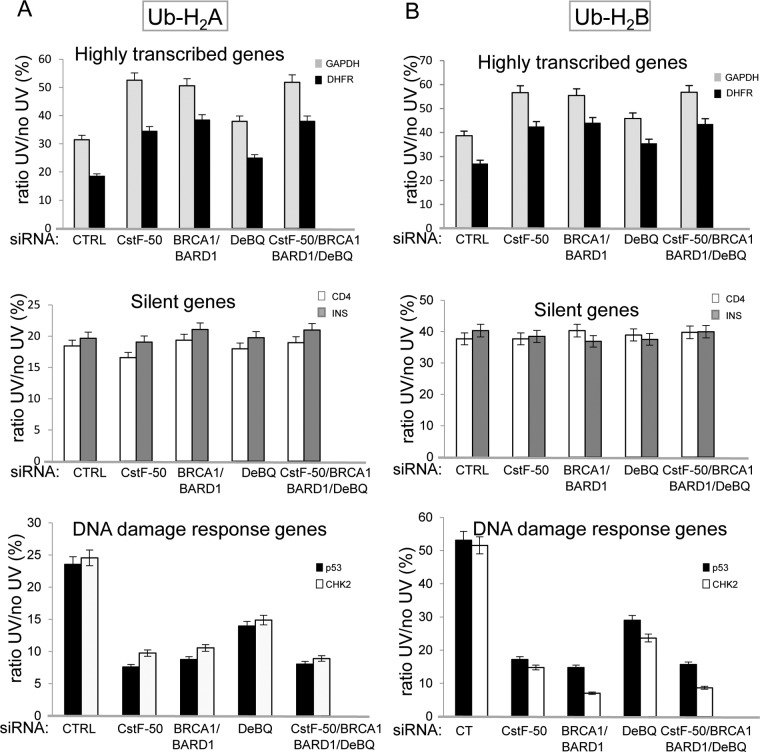
The content of ubiquitinated histones in the chromatin of differentially expressed genes changes in a BRCA1/BARD1- and CstF-50/p97-dependent manner during DDR. Chromatin extracts from HeLa cells treated with control, CstF-50, BRCA1/BARD1, or CstF50/BRCA1/BARD1 siRNA were prepared. The cells were also treated with UV irradiation (40 J m^−2^) and the p97 inhibitor DBeQ (10 μM) during the 2-h recovery. Chromatin extracts were immunoprecipitated with either Ub-H_2_A-specific (A) or Ub-H_2_B-specific (B) antibodies, followed by qPCR analysis of the immunoprecipitated DNA fragments using primers for differentially expressed genes. The error bars indicate standard deviations (*n* = 3).

Next, we further explored whether the effect of CstF-50-mediated activation of BRCA1/BARD1 E3 Ub ligase activity is evenly exerted throughout the genome. We tested the implications of BRCA1/BARD1- and CstF-50-mediated ubiquitination of histones on the chromatin structures of differentially expressed genes upon UV damage by performing chromatin immunoprecipitation (ChIP) assays, followed by quantitative PCR (qPCR). Briefly, chromatin extracts were prepared from cross-linked HeLa cells treated or not with UV and either control, CstF-50, or BRCA1/BARD1 siRNA (see Fig. S2C in the supplemental material). We also analyzed the effect of the concomitant depletion of BRCA1/BARD1/CstF-50 (triple knockdown) and inhibition of p97 ATPase activity by DBeQ. Samples were then immunoprecipitated with specific antibodies against either Ub-H_2_A or Ub-H_2_B (Millipore), and the immunoprecipitated DNA fragments were analyzed by qPCR using primers for differentially expressed genes. The primers were designed to amplify the transcribed region of each gene (see Fig. S2E in the supplemental material), since it was previously shown that ubiquitinated histones preferentially target this region (41). We tested highly expressed genes (encoding DHFR and GAPDH) (42–45), silent genes (encoding CD4 and insulin) (43), and DDR-related genes (encoding p53 and CHK2) (46). Sample normalization was done using nonimmunoprecipitated chromatin extracts. The immunoprecipitated chromatin values from UV-treated samples were lower than the ones from nontreated samples under all the conditions analyzed, reflecting the UV-induced decrease in the amount of ubiquitinated histones in the chromatin (Fig. 4). These results are consistent with previous studies describing the displacement of Ub-H_2_A (17) and Ub-H_2_B (35, 36) from UV-damaged chromatin.

Interestingly, depletion of BRCA1/BARD1 and CstF-50 expression slightly but reproducibly increased the ratio of immunoprecipitated chromatin values from UV-treated and untreated samples in highly expressed genes (Fig. 4A and B, top). This increase was observed when chromatin was immunoprecipitated with either Ub-H_2_A (Fig. 4A) or Ub-H_2_B (Fig. 4B) antibody. The concomitant knockdown of BRCA1/BARD1/CstF-50 resulted in a decrease similar to the one observed after the knockdown BRCA1/BARD1 and of CstF-50. Depletion of the expression of BRCA1/BARD1, CstF-50, or BRCA1/BARD1/CstF-50, as well as DBeQ treatment, did not change the content of either Ub-H_2_A or Ub-H_2_B in the chromatin of silent genes during DDR (Fig. 4A and B, middle). Strikingly, depletion of the expression of BRCA1/BARD1, CstF-50, or BRCA1/BARD1/CstF-50 or DBeQ treatment decreased the levels of both Ub-H_2_A and Ub-H_2_B in the chromatin of genes involved in DDR after UV treatment (Fig. 4A and B, bottom). Although, the mechanism involved in this differential effect needs further investigation, our results suggest that the BRCA1/BARD1/CstF-50 complex plays an important role in changing the chromatin structure of DDR genes to ensure efficient DNA repair and transcription of genes associated with this response. Together, our data indicate that the chromatin of differentially expressed genes shows different ubiquitinated H_2_A and H_2_B contents after DNA damage in a BRCA1/BARD1- and CstF-50-dependent manner, suggesting a specific mechanism of regulation of chromatin structure for each group of genes.

## DISCUSSION

BRCA1, in concert with BARD1, possesses significant E3 Ub ligase activity, which might account for many BRCA1/BARD1 roles in DNA repair, genome stability, and gene expression during DDR. Although a lot of work has been done to understand the biochemistry of the BRCA1/BARD1 E3 ligase, very little is known of its cellular targets, how these targets are chosen, the mechanism by which they are recruited to DNA damage sites, and how BRCA1/BARD1 E3 ligase activity is regulated during DDR. Here, we have provided insights into the roles of the mRNA processing factor CstF-50 and the Ub escort factor p97 in the regulation of BRCA1/BARD1 functions in the Ub pathway and chromatin structure remodeling during DDR. We showed that CstF-50 can interact not only with BRCA1/BARD1 (24), the escort factor p97 (Fig. 1A to C), and Ub (see Fig. S1A and B in the supplemental material) but also with some BRCA1/BARD1 substrates, such as RNAP II (24, 27), H_2_A, and H_2_B (Fig. 1G). Furthermore, CstF-50 and p97 have an additive effect on the activation of BRCA1/BARD1 autoubiquitination (Fig. 1D to F), BRCA1/BARD1-mediated polyubiquitination of RNAP II, and monoubiquitination of H_2_A and H_2_B (Fig. 1H to J). Our results also indicate that p97 has a nonenzymatic additive role in the E3 Ub ligase activity of BRCA1/BARD1. Our analysis of soluble nuclear (Fig. 2) and chromatin-bound (Fig. 3) extracts indicated that BRCA1/BARD1, CstF-50, and p97 are responsible, to a certain extent, for the polyubiquitination and degradation of RNAP IIO and the monoubiquitination of H_2_A and H_2_B and their consequent eviction from chromatin. Importantly, the content of Ub-H_2_A and Ub-H_2_B in the chromatin of differentially expressed genes changes during DDR in a BRCA1/BARD1- and CstF-50/p97-dependent manner (Fig. 4). Taken together, our studies indicate that the binding of CstF-50 to both BRCA1/BARD1 and its substrates might help, in cooperation with p97, in the assembly and/or stabilization of the ubiquitination complex during DDR, resulting in chromatin remodeling of genes differently transcribed during DDR.

**FIG 5:**
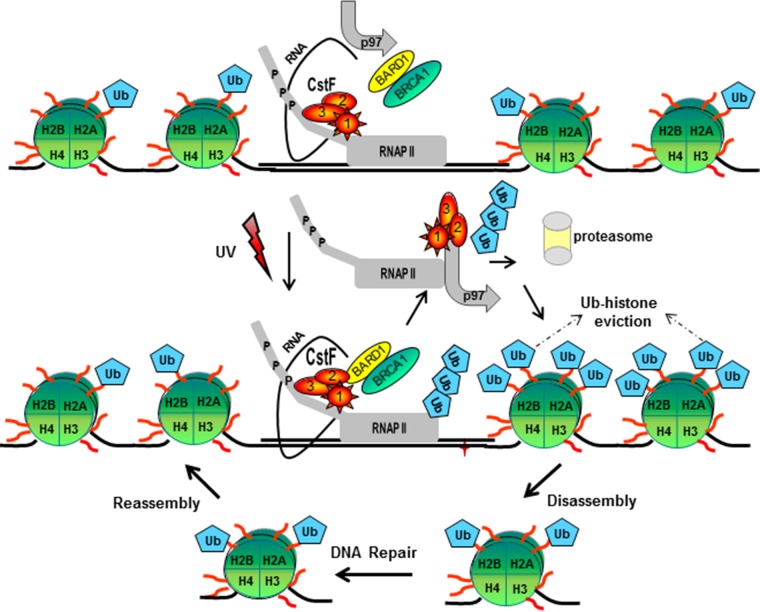
Model for the role of the polyadenylation factor CstF-50, the Ub escort factor p97, and the BRCA1/BARD1 Ub ligase during the progression of DDR. After exposure to UV treatment, CstF-50 associated with the elongating RNAP IIO recruits p97 and a BRCA1/BARD1-containing complex, inducing RNAP II and histone H_2_A and H_2_B ubiquitination. In that scenario, p97-mediated escorting allows the displacement of Ub-H_2_A, Ub-H_2_B, and poly-Ub–RNAP II from the chromatin, leading to the proteasome degradation of poly-Ub–RNAP II. The displacement of Ub-H_2_A and Ub-H_2_B allows the opening of the chromatin at the damage site, facilitating DNA repair. After DNA damage is repaired, chromatin structure reconstitution occurs. This is in agreement with the previously described “access-repair-restore” model (49).

Based on these results, we propose the following model (Fig. 5). Under nonstress conditions, normal levels of transcription and mRNA processing are observed due to the interactions of the elongating RNAP IIO and components of the mRNA 3′ processing machinery, specifically CstF-50 (6), and a chromatin structure that allows transcription showing low levels of monoubiquitinated H_2_A and H_2_B (reviewed in reference 30). After DNA damage, BRCA1/BARD1, as part of the RNAP II holoenzyme (47), associates with the escort factor p97 (18, 19) and senses sites of DNA damage and repair, and the inhibitory interaction with CstF-50 ensures that nascent RNAs are not erroneously polyadenylated at such sites, avoiding the expression of deleterious proteins (5). In that scenario, CstF-50 is involved in DNA damage-induced ubiquitination of RNAP II (9), which is an important event in DDR (48). At that point, chromatin must be locally destabilized to allow access of the repair machinery to the lesions (30, 49). The formation of the BRCA1/BARD1/CstF-50/p97 complex allows not only the UV-induced ubiquitination of RNAP IIO (9), which is an important event in DDR (48), but also monoubiquitination of H_2_A and H_2_B, which results in nucleosome remodeling by displacement of Ub-H_2_A (17) and Ub-H_2_B (35, 36) from UV-damaged chromatin and allows access of the repair machinery (49). In that scenario, the escort factor p97 drives the protein dynamic at chromatin by acting as a segregase that facilitates the extraction of substrate proteins (38), such as polyubiquitinated RNAP II, Ub-H_2_A, Ub-H_2_B, and the E3 Ub ligase complex.

After the repair is completed, the chromatin structure has to be restored to its original state. Consistent with the “access-repair-restore” model (49), the ubiquitination of H_2_A and H_2_B by BRCA1/BARD1/CstF-50 might represent a postrepair process that allows chromatin restoration (30, 50). Consistent with this, it has been shown that H_2_B ubiquitination interferes with chromatin compaction, leading to an open and biochemically more accessible chromatin conformation (51), and that H_2_A ubiquitination, a modification located on the opposite side of the nucleosomal surface, does not appear to hinder chromatin compaction (30). As the role of BRCA1/BARD1 in DDR might be ubiquitous and significant to different cell lines, such as neurons (52) and colon cancer cells (53), the model proposed here might be relevant to different cellular backgrounds.

Consistent with this, the segregase/escort activity of p97 might also lead to the UV-induced proteasome degradation of poly-Ub–RNAP II (6, 8, 54). p97 has been shown to facilitate the UV-dependent turnover of RNAP II at sites of stalled transcription in Saccharomyces cerevisiae (55) and mammalian cells (23). Verma and colleagues also showed that an adaptor for the CUL3 Ub ligase, UbX5, facilitates the degradation of chromatin-bound RNAP II. The formation of the BRCA1/BARD1/CstF-50 complex can also inhibit the mRNA 3′ processing machinery (5), allowing the elimination of prematurely terminated transcripts that could produce potentially deleterious proteins. Interestingly, the BRCA1/BARD1 complex interacts with RNAP IIO at sites of DNA damage, preferentially on active genes (56, 57). Consistent with this, it was reported that actively transcribed genes and silenced or repressed areas of the genome might require different chromatin-remodeling mechanisms for efficient repair (58).

Supporting this model, it has been reported that BRCA1/BARD1 plays a role in histone ubiquitination (11, 12, 14, 15, 29). BRCA1 deficiency results in a compromise chromatin structure that affects control of gene expression, most likely due to the loss of Ub-H_2_A and Ub-H_2_B. Other E3 Ub ligases, such as DDB1-2-CUL4B (16, 17), RING2 (31), RFN168 (59), RNF20/RNF40 (60), Bre1 (61), and RING1B (30, 39, 40), can ubiquitinate Ub-H_2_A and Ub-H_2_B under DNA-damaging conditions. Interestingly, p97 coordinates chromatin remodeling by binding to Cdc48, which is involved in H_2_B monoubiquitination with the cofactor Ubx3 (61). p97 has also been shown to facilitate the UV-dependent turnover of RNAP II, and an adaptor to CUL3 Ub ligase, UbX5, facilitates the degradation of chromatin-bound RNAP II (55). As CstF-50 binds to p97, helps BRCA1/BARD1 to recognize substrates, and facilitates the attachment of Ub to substrates, we cannot rule out the possibility that CstF-50 is a cofactor for BRCA1/BARD1 Ub ligase functions at the chromatin. Consistent with the function of CstF-50 as a BRCA1/BARD1 cofactor, p97 regulates the degree of ubiquitination of bound substrates in the presence of Ub-binding or substrate-processing cofactors that either activate or inhibit ubiquitination of the substrate (20, 62).

Our study provides evidence of a functional interplay of the mRNA processing factor CstF-50, the escort factor p97, BRCA1/BARD1 Ub ligase, and chromatin-bound proteins, such as H_2_A, H_2_B, and RNAP II. This interplay results in changes in the chromatin structures of differently expressed genes during DDR. As most of the factors and mechanisms involved in chromatin plasticity are poorly understood, the data presented here contribute to a better understanding of some aspects of chromatin dynamics during DDR.

## MATERIALS AND METHODS

### Tissue culture methods and DNA-damaging agents.

HeLa cells were cultured and treated with UV (40 J m^−2^), as described previously (7, 63). The cells were treated with 2 μM MG132 (Sigma) (6) and the p97 inhibitor DBeQ (10 μM; LifeSensors) as described previously (28).

### Purification of recombinant proteins.

Heterodimeric complexes comprised of full-length BARD1 (wild type [WT] and Q564H) and truncated BRCA1 (ΔBRCA1/BARD1) were generated as described previously (6, 64). Plasmids encoding His-Ub, His-UbcH5a, GST–CstF-50, and His-p97 were transformed into BL21 cells. His and GST fusion proteins were purified by binding to and elution from Ni-agarose and glutathione-agarose beads, respectively, as described previously (6).

### NE preparation.

After UV treatment, NEs were prepared from harvested cells as described previously (7, 63). The NEs were quickly frozen and stored at −80°C.

### Pulldown assays.

Interaction assays using His-Ub and GST–CstF-50 were performed as described previously (5). Equivalent amounts of pellets and supernatants were analyzed by immunoblotting. Pulldown assays (5) were carried out with ΔBRCA1/BARD1-Q564H, and the assay mixtures were washed with buffer A (1× phosphate-buffered saline [PBS]: 137 mM NaCl, 3 mM KCl, 10 mM Na_2_HPO_4_, 1.8 mM KH_2_PO_4_, 0.01% Nonidet P-40, and 0.04% bovine serum albumin) including 300 mM NaCl. The GST-Q564H BARD1 mutant was obtained and expressed as previously described (5, 24).

### IP and Western blot analysis.

Total protein (100 μg) from the indicated NEs was immunoprecipitated as described previously (7, 63). Immunoprecipitations (IPs) were performed with antibodies against CstF-50 (Bethyl), Ub (P4D1; Santa Cruz Biotechnology), HA (conjugated beads from Sigma), and p97 (Bethyl). Western blot analysis was done with antibodies against histones (MAB052; Millipore), RNAP IIO (H5; Covance), BRCA1 (sc-1021; Santa Cruz Biotechnology), BARD1 (H-300; Santa Cruz Biotechnology), Ub-H_2_A (Millipore), Ub-H_2_B (Millipore), and Topo II (Santa Cruz).

### CstF-50, BRCA1/BARD1, and RING1B siRNA knockdown.

On-Targetplus Smartpool siRNAs specific for human CstF-50, BRCA1/BARD1, RING1B, and control RNA duplex were synthesized by Dharmacon RNA Technologies (Lafayette, CO). Using a pool of siRNAs that target different sites in the message and a combination of sense strand inactivation with region modifications prevented off-target effects. siRNA and UV treatments (40 J m^−2^) were as described previously (7, 63). HeLa cells were transfected with Lipofectamine RNAiMax (Invitrogen). BRCA1/BARD1 double knockdown was performed as described previously (6).

### Ubiquitination reactions.

Autoubiquitination reactions were conducted as described previously (6). The reactions were performed with 1 μg of His-Ub, 0.25 μg of UbcH5c, 20 ng of ΔBRCA1/BARD1 heterodimer, and increasing amounts of GST–CstF-50. Alternatively, 1 μg of commercially available histones (New England BioLabs) or 100 ng of purified RNAP IIO (26) was added to the ubiquitination reaction mixtures, and conditions were as described previously (29). The HeLa cells were transfected with HA-Ub and FLAG-histone expression vectors, together with either control or CstF-50 siRNA as indicated previously. Whole-cell extracts were prepared, followed by sonication, and immunoprecipitated with either anti-HA-conjugated beads (Sigma) or anti-FLAG M2 magnetic beads (Sigma) following the manufacturer's instructions. Equivalent amounts of pellets and supernatants were analyzed by immunoblotting.

### ChIP assays.

Chromatin was prepared from HeLa cells treated under the different conditions described in the figure legends using a Magna-EZ ChIP kit (Millipore) and following the manufacturer's instructions. IP was carried out using ChIP grade Ub-H_2_A and Ub-H_2_B antibodies (Millipore). The Ub specificity of these antibodies had been previously validated (65, 66).

### qPCR assays.

Equal amounts of DNA from ChIP samples were quantitatively amplified using Power SYBR green PCR master mix (Applied Biosystems) with primers for differentially expressed genes. Relative levels were calculated using the Δ*C_T_* method.

### Cellular fractionation.

Chromatin-bound fractions were prepared from HeLa cells using a cell fractionation kit (Pierce), following the manufacturer's instructions.

## Supplementary Material

Supplemental material
