# Supplementary material for: mRNA Processing Factor CstF-50 and Ubiquitin Escort Factor p97 Are BRCA1/BARD1 Cofactors Involved in Chromatin Remodeling during the DNA Damage Response
Source: Mol Cell Biol. 2018 Jan 29;38(4):e00364-17. doi: 10.1128/MCB.00364-17 (PMC5789026; doi:10.1128/MCB.00364-17)
Supplement: Supplemental material [file supp_38_4_e00364-17__index.html]

mRNA Processing Factor CstF-50 and Ubiquitin Escort Factor p97 Are BRCA1/BARD1 Cofactors Involved in Chromatin Remodeling during the DNA Damage Response — Supplemental material 

# mRNA Processing Factor CstF-50 and Ubiquitin Escort Factor p97 Are BRCA1/BARD1 Cofactors Involved in Chromatin Remodeling during the DNA Damage Response

## Supplemental material

- Supplemental file 1 -

  Fig. S1 (Blots) and S2 (Blots and primers)

  PDF, 377K
