# Supplementary material for: mRNA Processing Factor CstF-50 and Ubiquitin Escort Factor p97 Are BRCA1/BARD1 Cofactors Involved in Chromatin Remodeling during the DNA Damage Response
Source: Mol Cell Biol. 2018 Jan 29;38(4):e00364-17. doi: 10.1128/MCB.00364-17 (PMC5789026; doi:10.1128/MCB.00364-17)
Supplement: Supplemental material [file MCB.00364-17_zmb999101696s1.pdf]

Fonseca et al. Supplemental Fig. 1

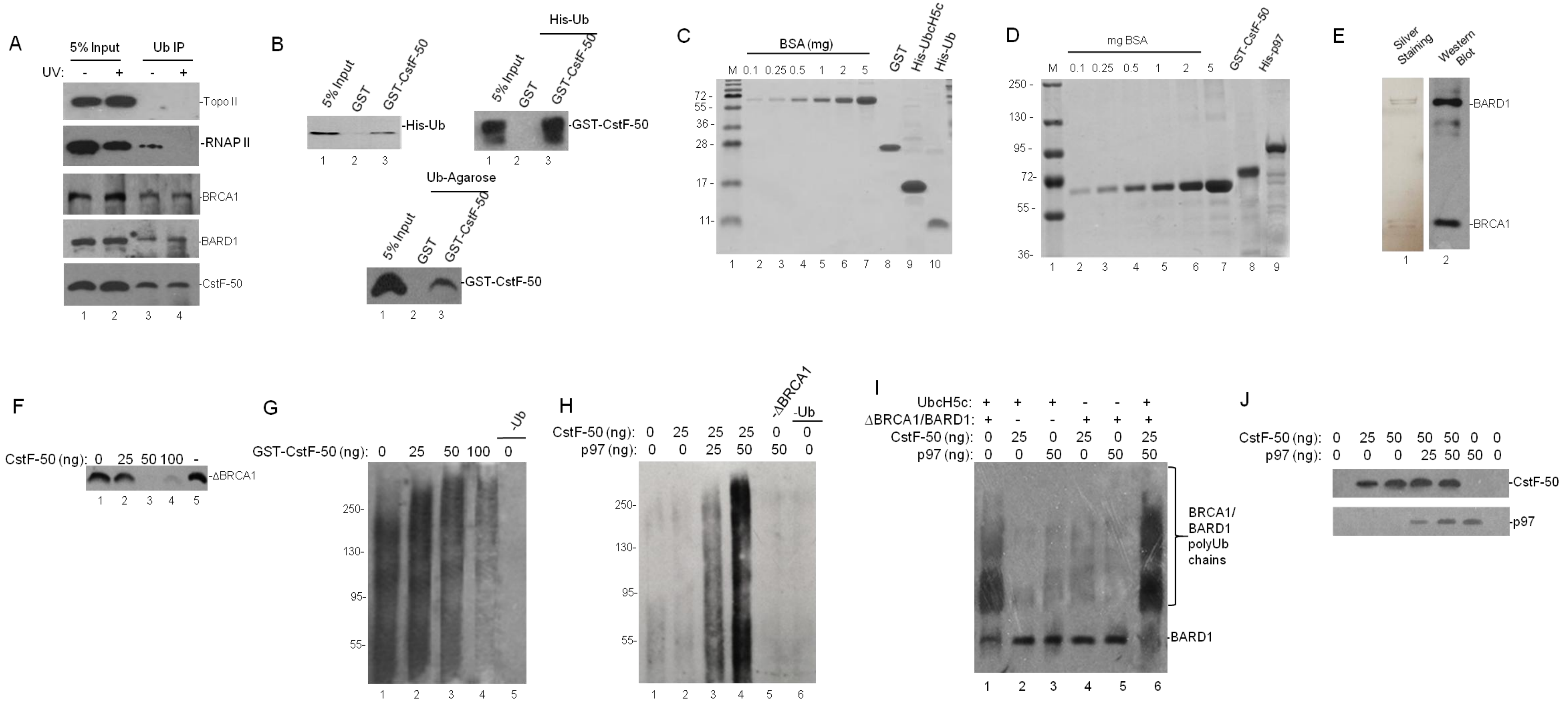

Figure S1: A) Ub, CstF-50, BARD1, BRCA1 and RNAP II co-IPed from NEs of HeLa cells. NEs of cells exposed to UV irradiation (40 Jm^{-2}) and allowed to recover for 2 h were prepared as in Fig. 1A, and IPed with anti-Ub. Equivalent amounts of the pellets (IP) were resolved by SDS-PAGE and proteins were detected by immunoblotting using the indicated antibodies. 5% of the NE used in the IP reaction is shown as input. A representative IP reaction from three independent assays is shown. B) CstF-50 interacts directly with Ub. Immobilized GST-CstF-50 or GST (left panel) on glutathione-agarose beads was incubated with 1 μg of His-Ub. Immobilized His-Ub on nickel beads (right panel) or Ub-agarose (lower panel) was incubated with GST-CstF-50 or GST. Bound proteins were eluted, resolved in SDS-PAGE and equivalent amounts of the pull-downs (PD) were analyzed by immunoblotting with either anti-Ub or anti-CstF-50. 5% of His-Ub or GST-CstF-50 used in the reaction is shown as input. Recombinant proteins were treated with RNase A. Representative pull-down reactions from three independent assays are shown. C-D) Purified recombinant proteins for His-Ub, His-UbcH5c, His-p97, GST, and GST-50 were separated in PAGE and quantified with bovine serum albumin (BSA) by staining with Coomassie Blue. E) Purified His-BARD1/ΔBRCA1 was separated by PAGE and analyzed by Silver staining and Western blot with antibodies against BRCA1 and BARD1. F) Anti-BRCA1 blot from *in vitro* ubiquitnation reactions shown in Fig 1d. G-H) Blots of the *in vitro* ubiquitination reactions shown in Fig. 1d and e, respectively, immunoblotted with anti-Ub antibodies. I) *In vitro* ubiquitination reactions were carried out as described in Fig 1(d) as control for the inability of CstF-50 to function as E2 or E3 Ub ligase. J) Representative immunoblots showing the increasing amount of GST-CstF-50 and His-p97 in the ubiquitination reaction corresponding to Fig 1J.

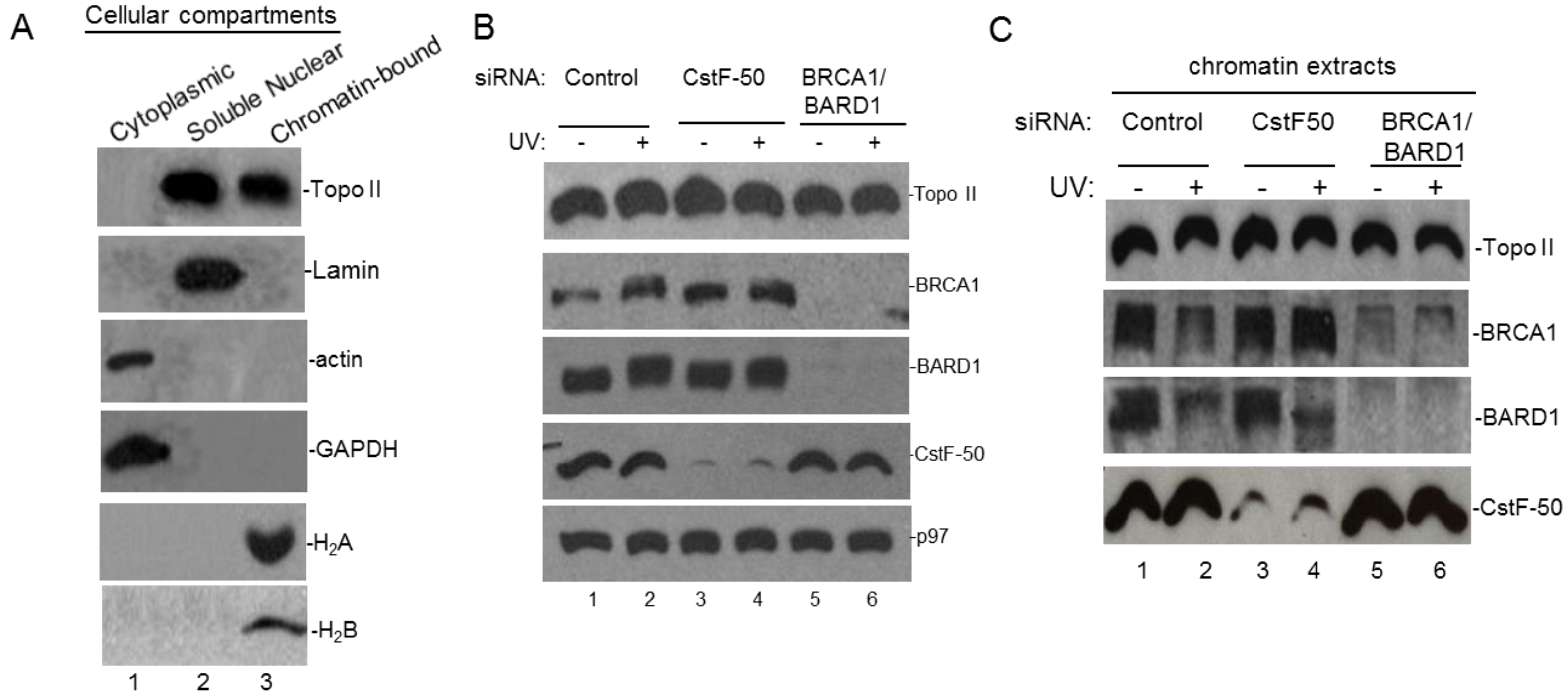

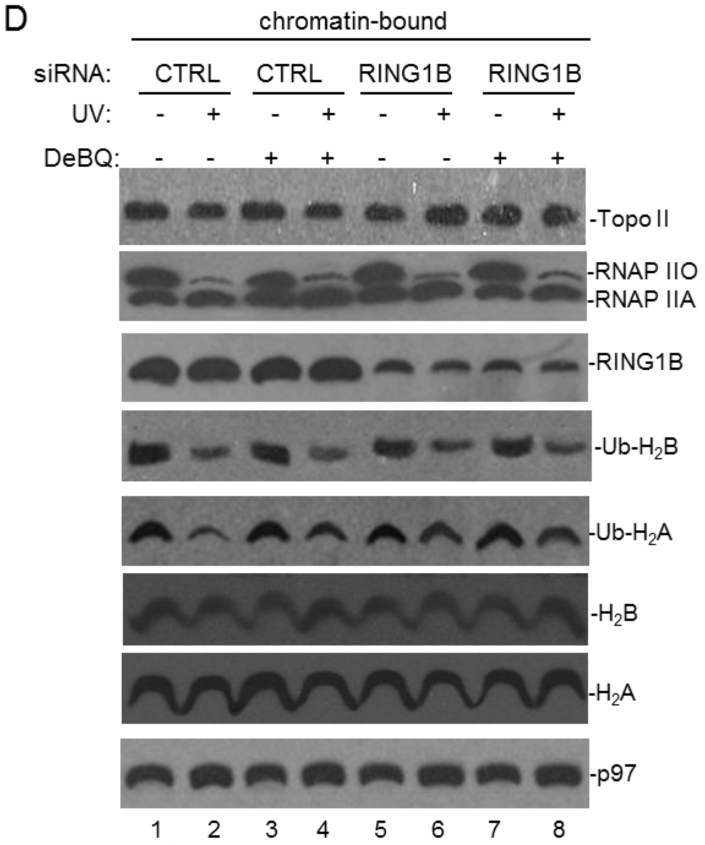

E

Gene	Forward Primer	Reverse Primer
GAPDH	5'-CTAGGCAGCAGCAAGCATTC-3'	5'-CCCAACACCCCCAGTCATAC-3
DHFR	5'-AATTCCATCGGCAGTCCTCA-3'	5'-GGTGGGGCTTAGATTAGGCA-3
Insulin	5'-CCTGTAGGTCCACACCCAGT-3'	5'-GGAGGACACAGTCAGGGAGA-3
CD4	5'-TAGGCGACAGAGCAAGACTC-3'	5'-GTGTGTGTGTGTGTGTGCTT-3
p53	5'-AGTGTCCGAAGAGAATGGGC-3'	5'-CACCCTTCCCCACCTGATAC-3
CHCK2	5'-GCTCACTGTGGCCTTGATCT-3'	5'-TAAGGCAGGAGGATGGGTTG-3'

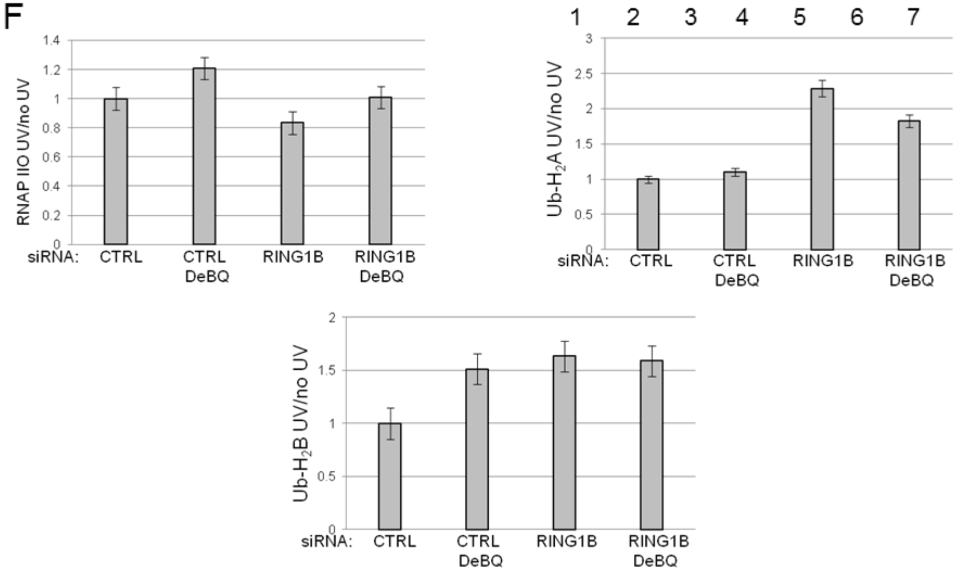

Figure S2: A) HeLa cells were used in a subcellular fractionation kit (Pierce) to release the protein content of each cellular compartment, as instructed by the manufacturer. Equivalent amounts of total protein content from each fraction were analyzed by SDS-PAGE. Antibodies against topoisomerase II (Topo II), lamin, glyceraldehyde-3-phosphate dehydrogenase (GAPDH), actin, and histone H_2A and H_2B were used as controls for each fraction. B) Representative immunoblot of soluble NEs from Hela cells transfected with either control, CstF-50 or BRCA1/BARD1 siRNA to confirm the knockdown efficiency. These samples were used in Fig. 2. B) Representative immunoblot of chromatin fractions from Hela cells transfected with either control, CstF-50 or BRCA1/BARD1 siRNA to confirm the knockdown efficiency. These samples were used in Fig. 4. C) Chromatin-bound fractions were prepared from HeLa cells treated with either control or RING1B siRNAs and analyzed by Western blot with the indicated antibodies. Cells were also exposed to UV (40 Jm^{-2}) and DeBQ (10 μM) treatment during the 2 h recovery. D) PCR primers of differentially expressed genes used in the ChIP/qPCR analysis shown in Fig. 4. E) Quantification of the blots shown in d).
